# Phrenology Examined

**Published:** 1846-03

**Authors:** 


ART. IV.—*Phrenology Examined.* By P. FLOURENS, Member of the French Academy, Perpetual Secretary of the Royal Academy of Sciences, (Institute of France), Member of the Royal Societies of London and Edinburg, of the Royal Academy of Sciences of Stockholm, of Munich, and of Turin, &c. &c. Professor of Comparative Physiology at the Natural History Museum at Paris. Translated from the Second Edition of 1845, by CHARLES DE LUCENA MEIGS, M. D., Memb. Amer. Phil. Soc., &c. &c. Philadelphia: Hogan & Thompson. 1846. (16mo. pp. 144.)

Phrenology, having long bestrode the earth "like a Colossus," has, at last, met an assailant in one of the first anatomists and physiologists of the age. The foeman is worthy of the steel of Gall himself, if he could return from the grave. M. Flourens is distinguished even among the savans of the French metropolis, and he denounces phrenology as "a bad philosophy." We are phrenologists; but we desire the truth. If we have been confiding in an unsound doctrine, let us be made to see our error, and we shall renounce it. It is but fair, at least, to M. Flourens, as it is due to truth, that his objections should be heard and pondered.

The ground-work of his examination is Gall's great work on the Anatomy and Physiology of the nervous system, the fundamental propositions of which are, that the understanding resides exclusively in the brain, and that each particular faculty of the understanding is provided in the brain with an organ proper to itself. "Now," says M. F., "of these two propositions, there is certainly nothing new in the first one, and perhaps nothing true in the second one."

This is strong ground, but, as to the first proposition, it is unquestionably true. It was no discovery of the great phrenologist, that the brain is the organ of the mind; such a claim was never set up by him. This being settled, then, we go on at once to the arguments in support of the second proposition; namely, that there is nothing new in the science of organology and we shall give the arguments in the author's own language. He states the phrenological doctrines thus:

"Gall's philosophy consists wholly in the substitution of *multiplicity* for *unity*. In the place of one general and single brain, he substitutes a number of small brains: instead of one general sole understanding, he substitutes several individual understandings. These pretended *individual understandings* are the *faculties.*

"Now, Gall admits the existence of twenty-seven of these faculties, each one of them (since each one is a peculiar understanding) endowed with its perceptive faculty, its memory, its judgment, its imagination, &c.

"Hence, there are twenty seven perceptive faculties, twenty-seven memories, twenty-seven judgments, twenty-seven imaginations, &c.

"Hence there can be nought but the faculties; and, according to Gall, these faculties are so distinct, that he attributes to each particular one a separate organ. He divides the understanding into little understandings."

Upon which Flourens remarks—

"Under the title of *fundamental faculties,* Gall confounds all things together—the passions, the instinct, the intellectual faculties. These faculties, which are at the basis of his whole philosophy, he knows not even how to denominate them. He calls them instincts, inclinations, senses, memories, &c. There is a memory or sense of things, a memory or sense of persons, &c He confounds the instinct that leads certain animals to live in elevated regions with pride, which is a moral sentiment in man; the carnivorous instinct with courage; he believes that conscience, (which is the soul judging itself,) is nothing but a modification of a particular sense, the sense of benevolence, &c.

"The hesitation of his mind is visible every where.

"'I leave it to the reader,' says he, 'to decide whether the fundamental faculty to which this penchant relates, should be denominated senses of elevation, self-esteem,' &c,

"'To speak correctly,' continues he, 'firmness is neither a penchant nor a faculty; it is a mode-of-being, which gives to a man a distinctive quality, which is called character.'

"Finally, he writes the following paragraph, perhaps the most singular one that he ever wrote, for it shows in the clearest manner how little confidence he had in his own psycology.

"'If we are materialists because we do not admit the existence of a unit-faculty of the soul, but recognise several primitive faculties, we ask whether the ordinary division of the faculties of the soul into understanding, will, attention, memory, judgment, imagination, and affections and passions, expresses nothing more than a primitive unit-faculty? If it be asserted that all these faculties are merely modifications of a sole and same faculty, what can hinder us from making the same assertion as to the faculties whose existence we do admit.'

"To be sure, nothing prevents you. Or rather every thing constrains you to do so. There is therefore one sole faculty, of which all the other faculties are but moods. You return then to the common philosophy, and consequently you no longer possess a peculiar philosophy.

"The problem proposed by Gall is at the same time physiological, psycological, and anatomical.

"In our first article an account has been given of Gall's *physiology*, and it has been shown to be generally disproved by direct experiment. In the present one his *psycology* has been examined, and it is confuted by the consciousness (*le sens intime*). It only remains for us now to examine his *anatomy*."

The estimate put by Flourens upon the anatomy of Gall is not very high. He says:

"The truth is: Gall never had any settled opinion upon what he called the organs of the brain; he never saw those *organs*, and he imagined them for the use of his *faculties*. He did what so many others have done. He commenced with imagining a hypothesis, and then he imagined an anatomy to suit his hypothesis.

"But as to the pretended *organs of the brain*, are they really situated at the surface of the brain, as Gall asserts? In plain terms, is the surface of the brain the only active part of the organ? Here is a physiological experiment that shows how very much mistaken Gall is.

"You can slice off a considerable portion of an animal's brain, either in front, behind, on one side, or on the top, without his losing any one of his faculties.

"The animal may, therefore, lose all that Gall calls surface of the brain, without losing any of his faculties. Therefore it cannot be that the organs of the faculties reside at the *surface of the brain.*

"And comparative anatomy is not less opposite to Gall's opinions than is direct experiment itself. I shall not follow him here in the detail of his localizations. How could these localizations have any meaning? He does not even know whether an organ is a *fascicle of fibres*, or a *fibre*.

"For example; he places what he calls the instinct of propagation in the cerebellum, and what he calls the *instinct of the love of offspring*, in the posterior cerebral lobes; and he looks upon these two localizations as the very surest in his book.

"'I should wish,' says he, 'that all young naturalists might begin their researches with the study of these two organs. They are both easily to be recognised,' &c.

"What! The cerebellum, so different in its structure from the great brain, is the cerebellum, like the brain, to be considered an organ of instinct? And what is more, is it to be regarded as the organ of a single instinct only, while the brain shall have twenty-six of them?

"I have already said that the cerebellum is the seat of the principle that presides over the locomotion of the animal, and that it is not the seat of any instinct.

"Gall places the love of offspring in the posterior lobes of the brain. Now, the love of offspring, and especially maternal love, is every where to be found among the superior animals; it is found in all the mammifera, in all the birds. The posterior lobes of the brain, therefore, ought to be found in all these beings. Not at all: the posterior lobes are wanting in most of the mammifera; they are wanting in all the birds.

"Gall locates the faculties that are common to both man and animals, in the posterior part of the brain; in the anterior part he places those that are peculiar to man alone. According to this plan, the most *persistent* portion of the brain will be the posterior portion, and the least persistent the anterior portion. But the inverse of the proposition holds. The parts that are most frequently wanting are the *posterior parts*, and those that are most invariably present are the *anterior parts*."

If, he says, from the brain, we pass on to consider the cranium, "all the foregoing is found to be of still greater force." The cranium, Gall himself admitted, represents the superficial configuration of the brain but very imperfectly. The author wonders that, in view of this, Gall should have ventured to "inscribe his twenty-seven faculties upon the skulls." He quotes a cruel remark from Vimont, an able anatomist and decided phrenologist, that "Gall's work is fitter to lead into error than to give a just idea of the seats of the organs." Still, he adds, "this same M. Vimont inscribes twenty-nine names on the skull of a goose."

The author states his own views respecting the functions of the brain in the following passage:

"The brain, en masse, the *encephalon*, is then a multiple organ; and this multiple organ consists of four particular organs: the cerebellum, the seat of the principle that regulates the movements of locomotion; the tubercula quadrigemina, seats of the principle that regulates the sense of sight; the medulla oblongata. in which resides the principle that determines the respiratory motions; and the brain proper, the seat, and the exclusive seat of the intelligence.

"Therefore, when the phrenologists promiscuously place the intellectual and moral faculties in the brain, considered en masse, they deceive themselves. Neither the cerebellum, the quadrigeminal tubercles, nor the medulla oblongata can be regarded as seats of these faculties. All these faculties dwell solely in the brain, properly so called, or the hemispheres.

"The question as to the precise seat of the intelligence, has undergone a great change since the time of Gall. Gall believed that the intelligence was seated indifferently in the whole encephalon, and it has been proved that it resides only in the hemispheres.

"Further, it is not the encephalon taken en masse that is developed in the ratio of the intelligence of the creature, but the hemispheres. The mammifera are the animals most highly endowed with intelligence; they have, other things being equal, the most voluminous hemispheres. Birds are the animals most highly endowed with power of motion; their cerebellum is, other things being equal, the largest. Reptiles are the most torpid and apathetic of animals; they have the smallest brain, &c.

"Every thing concurs then to prove, that the encephalon, in mass, is a multiple organ with multiple functions, consisting of different parts, of which some are destined to subserve the locomotive motions, others to motions of the respiration, &c., while one single one, the brain proper, is designed for the purposes of the intellection.

"This being conceded, it is evident that the entire brain cannot be divided, as the phrenologists divide it, into a number of small organs, each of which is the seat of a distinct intellectual faculty; for the entire brain does not serve the purposes of what is called the intelligence. The hemispheres alone are the seats of the intellectual power; and consequently, the question as to whether the organ, the seat of the intelligence may be divided into several distinct organs, is a question relative solely to the uses and powers of the hemispheres."

He adds—

"It has been shown by my late experiments, that we may cut away, either in front, or behind, or above, or on one side, a very considerable slice of the hemisphere of the brain, without destroying the intelligence. Hence it appears, that quite a restricted portion of the hemispheres may suffice for the purposes of intellection in an animal.

"On the other hand, in proportion as these reductions by slicing away the hemispheres are continued, the intelligence becomes enfeebled, and grows gradually less; and certain limits being passed, is wholly extinguished. Hence it appears that the cerebral hemispheres concur, by their whole mass, in the full and entire exercise of the intelligence.

"In fine, as soon as one sensation is lost, all sensation is lost; when one faculty disappears, all the faculties disappear. There are not, therefore, different seats for the different faculties, nor for the different sensations. The faculty of feeling, of judging, of willing any thing, resides in the same place as the faculty of feeling, judging, or willing any other thing, and consequently this faculty, essentially a unit, resides essentially in a single organ."

These objections are weighty, but do not appear to us fatal to phrenology. They, who are doing most to shake public faith in the science, are the phrenologists themselves. Years ago we read the works of Gall, and were told that his chart of the cranium was a true mapping of the brain. Spurzheim afterwards made certain modifications in his chart, introducing new organs, and changing the seats of old ones; and then we were assured that the truth of the cranioscopy was no longer a matter of doubt. It was declared a *certain* science; more exact than chemistry. Vimont now warns us against the fallacies of Gall's chart, assuring us, that it is better calculated to mislead than to give a just idea of the seats of the organs. And worse than all, the *neurologists* have been overhauling the system, and have so changed its aspect, that Gall would scarcely recognise it as the same. Few organs have escaped the revision of these philosophers, or been allowed to stand where they were first set down; while the number has swelled from twenty-seven to more than two hundred, and is still increasing. They cover, now, not only the front, apex, and rear of the head, but the face, and the neck, and are so crowded that, as remarket by Leuret of the mapping on the head of a goose, "it would be a marvellous thing to be able to write their names upon the brain, and a greater marvel to discover them." Amid these revolutions, following each other in such quick succession, faith is staggered, and we know not what to believe. Doubtless, much error has been mixed up with the truth, and the system is still imperfect. New charts must be made, as multiplied observations show the mistakes of former phrenologists. But we cannot agree with our eminent friend, the translator, "that Gall was wholly mistaken in his views of the human mind; and of course, that all the cranioscopists, mesmerizers, and diviners who have followed his track, or risen upon the basis of his opinions, are equally in error.' Along with the tares there is good grain coming to maturity—not, perhaps, the abundant harvest to which we once looked forward—but still beautiful and promising plants which will prove a blessing to the nations.

